# LEAFY COTYLEDON 2: A Regulatory Factor of Plant Growth and Seed Development

**DOI:** 10.3390/genes12121896

**Published:** 2021-11-26

**Authors:** Boling Liu, Ge Sun, Changju Liu, Shijuan Liu

**Affiliations:** School of Life Science, Qufu Normal University, Qufu 273165, China; lblzzkk@126.com (B.L.); sungemakang@163.com (G.S.); lcjsmkx@163.com (C.L.)

**Keywords:** embryogenesis, somatic embryogenesis, plant growth, seed development, transcription factor, *LEC2*

## Abstract

Transcription factors are key molecules in the regulation of gene expression in all organisms. The transcription factor LEAFY COTYLEDON 2 (LEC2), which belongs to the DNA-binding protein family, contains a B3 domain. The transcription factor is involved in the regulation of important plant biological processes such as embryogenesis, somatic embryo formation, seed storage protein synthesis, fatty acid metabolism, and other important biological processes. Recent studies have shown that *LEC2* regulates the formation of lateral roots and influences the embryonic resetting of the parental vernalization state. The orthologs of *LEC2* and their regulatory effects have also been identified in some crops; however, their regulatory mechanism requires further investigation. Here, we summarize the most recent findings concerning the effects of LEC2 on plant growth and seed development. In addition, we discuss the potential molecular mechanisms of the action of the *LEC2* gene during plant development.

## 1. Introduction

Seeds are the key means by which terrestrial plants adapt to changing environments in the processes of evolution and diversification, and seed development occurs after zygotic embryogenesis. When seeds are in the maturation stage of growth and development, energy reserves continue to accumulate, while the seeds gain desiccation tolerance [1]. This process is strictly controlled at the transcription level, involving the AFL (ABI3/FUS3/LEC2) (ABA INSENSITIVE3/FUSCA3/LEAFY COTYLEDON 2) subfamily of B3 transcription factors (TFs). Researchers have shown that the B3 TF gene family developed within the green algae family 1200–725 million years ago, and the genes are present in all photosynthetic organisms [2]. AFL members interact with LEC1 (LEAFY COTYLEDON1) and LEC1-LIKE, which belong to the CCAAT-binding factors of the HAP3 family to control seed growth and development [3,4,5,6]. These genes have been collectively named L-AFL [7].

Members of the L-AFL family are considered to be the key regulatory TFs during the seed maturity stage [8]. The LEC2 TF establishes an ideal cellular environment for the formation of the zygotic embryo and its later stages of development [9,10]. Early embryonic development is the main period of expression of *LEC2*; at times, it is also expressed in vegetative organs [11,12]. LEC2 contains two domains named B2 and B3 [13]. It appears that the plant-specific B3 domain encoded by the *LEC2* gene recognizes the conserved RY motif to transcriptionally regulate the expression of zygotic embryogenesis-specific genes and that it promotes somatic embryo formation at the maturation stage [12,14,15]. The *LEC2* gene could be directly repressed by E2FA binding to an E2F-binding site during the seed maturation phase [16]. A ChIP assay suggested that PHABULOSA acts directly on the LEC2 promoter during embryogenesis [17]. An analysis using reporter genes indicated that LEC2 is negatively regulated by miRNA pathways during early embryogenesis [18]. miRNA is responsible, directly or indirectly, for repressing LEC2 in the embryo until it is required [17,18,19]. Retinoblastoma-related proteins facilitate seedling establishment by directly or indirectly repressing the promoters of late embryogenesis genes, including LEC2, during seed germination [20].

*LEC2* participates in a variety of signaling pathways and regulates the expression of numerous crucial genes during the growth and development of plants. Early studies have shown that the mutation of LEC2 in *Arabidopsis thaliana* altered the morphology of the embryo and caused certain local defects in the seed protein stockpile and its desiccation tolerance [12,21]. In addition, *LEC2* mutations halted the ability of somatic embryos to emerge from *A. thaliana* explants [22]. Through further in-depth exploration of the biological functions of *LEC2*, it was shown that the ectopic expression of *LEC2* caused the accumulation of seed storage lipids and proteins in plant nutritive organs [23,24], further inducing vegetative cells to form somatic embryos without exogenous auxin or seed-specific genes being expressed in the leaves [12,23]. In *A. thaliana*, the function of *LEC2* has been explained in terms of many aspects (Table 1).

Although major advances have been made concerning *LEC2*, its regulatory mechanism at the cellular and molecular levels remains unclear. Here, we discuss the impact of *LEC2* on the growth and development of plants.

## 2. The Effect of *LEC2* on Plant Early Embryo Morphogenesis

The development of seeds can be loosely divided into two stages: early embryonic development and maturation. In general, plants achieve desiccation tolerance by accumulating stored materials at the later stage of seed maturity. Embryogenesis is an important stage in the development of higher plants, and the LEC2 TF plays a pivotal regulatory role in controlling embryogenesis in *Arabidopsis*.

In the process of plant early embryogenesis, specialized leaves called cotyledons are produced. Compared with ordinary leaves, these embryonic leaves have large differences in morphology and gene expression patterns. When *AtLEC2* is mutated, the cotyledons undergo certain changes, including rounding in shape and the development of abnormal protrusions on the surface. Mutant cotyledons produce trichomes characteristic of leaves, indicating that *AtLEC2* is important for maintaining cotyledon traits during early embryogenesis [32].

Recent research has shed new light on *AtLEC2*’s involvement in the development of early embryos. When plants undergo a long winter, the polycomb protein silences the potent flower repressor *FLOWERING LOCUS C* (*FLC*) that induces flowers to undergo vernalization. *VIVIPAROUS1/ABI3-LIKE1* (*VAL1*) and *VAL2* are necessary for this process [33,34]. LEC2 and FUS3 are also required for embryonic FLC reactivation in early embryos following parental vernalization [30]. Late flowering is dependent on FLC in non-vernalized plants. However, this phenomenon is suppressed in *LEC2* and *FUS3* seeds. Hence, LEC2 and FUS3 are involved in embryonic FLC reactivation. FLC reactivation is also suppressed by *LEC2* in vernalized seedlings. In addition, the parental vernalization of T_2_ progeny from *FUS3* plants caused a reduction in FLC expression in the seedlings of T_3_ progeny [30]. LEC2 and FUS3 bind to the cold memory element of *FLC* to reactivate FLC expression in early embryos following parental vernalization. The ectopic induction of LEC2 or FUS3 activity can antagonize FLC repression mediated by VAL1 and VAL2 in seedlings. The B3 domain TFs LEC2 and FUS3 can replace VAL1 and VAL2 to reverse the chromatin-mediated silencing of FLC by polycomb proteins, thereby preventing the enrichment of histone 3 lysine 27 trimethylation and eliminating the parental retention of winter cold memory during early embryogenesis [30].

## 3. The Effect of *LEC2* on the Maturation of Plant Seeds

In the maturation stage after embryogenesis, certain storage products are accumulated during the seed filling process in order for growth to be restored under favorable environmental conditions [35,36]. The seed has three different regions: the filial embryo, the filial endosperm, and the maternal seed coat, which have major differences in terms of their genotypes [37]. In various plant species, fatty acids, sugars, starch, and storage proteins accumulate in the endosperm or embryo of the seeds [38]. *LEC2* imparts a regulatory effect on the formation of storage compounds during plant seed development. Studies have shown that FUS3 in the L-AFL family could inhibit the expression of *GA3ox1* and *GA3ox2* (GA biosynthesis genes) [39,40], while *LEC2* could activate *LEC1* and *FUS3* genes to induce embryo maturation [24]. In addition, *LEC2* directly induces *AGL15* (*AGAMOUS-LIKE15*) [14], and *AGL15* regulates the expression of the GA-related genes *GA3ox2* and *GA2ox6* [41,42]. These findings indicate that *LEC2* regulates genes that are related to GA biosynthesis to affect the embryonic maturation stage of seeds.

In *A. thaliana*, the main storage compounds of seeds are lipids and seed storage proteins (SSP) [43]. Studies of the regulation of gene expression in plants have demonstrated that SSP is strictly regulated in time and space. Previous research has shown that *AtLEC2* regulates the expression of *SSP* genes. *At2S3* is a storage protein gene. Through yeast hybrid screening, the TFs LEC2 and FUS3 were shown to directly activate the expression of the *At2S3* promoter and regulate it in a partially redundant manner [11]. Moreover, LEC2 also has a regulatory effect on fatty acid metabolism, mainly because it could influence the WRINKLED1 (WRI1) factor that encodes the transcription of fatty acids [44].

*LEC2* is preferentially expressed during seed maturation. The mutation of the *LEC2* gene in *A. thaliana* resulted in reductions in protein and oil content by 15% and 30%, respectively, whereas sucrose and starch content were sharply increased by 140% and 500% relative to the wild type [25]. For the phenomenon of increased sucrose, studies have shown that the sucrose synthase (SUS) gene in *A. thaliana* is regulated by *LEC2*. SUSs play a central role in carbon metabolism in plant heterotrophic tissues. Among these, *AtSUS2* (*At5g49190*) and *AtSUS3* (*At4g02280*), members of the SUS gene family, are upregulated in *A. thaliana* seeds [45,46,47,48]. During the growth and development of the seeds, the contents of *AtSUS2* and *AtSUS3* gradually accumulate when *AtLEC2* is mutated. This result indicates that *AtLEC2* has an epistatic effect on the two sucrose synthase genes [49].

**Figure 1:**
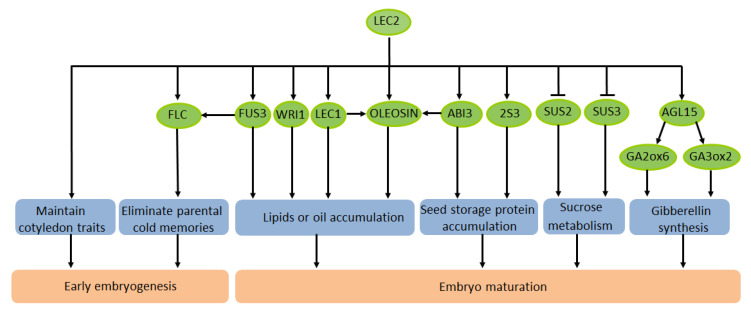
Schematic diagram of the transcription factor LEC2 regulating seed development of *A. thaliana*.

There has been some progress in understanding the mechanism of *LEC2* in the regulation of lipids and oil in seeds. Lipids and oils extracted from plants are extremely important renewable bioenergy materials. In plants, the main component of oil in seeds is triacylglycerol (TAG), and its synthesis can be enhanced by artificial modification. Meanwhile, the synthesis of fatty acids can also be artificially regulated. A recent study discovered an interesting phenomenon in *A. thaliana*: *LEC2* could trigger the stockpiling of oil in leaves and seed-specific mRNA. OLEOSIN, the main structural protein of oil bodies during seed development, is highly expressed in seeds [50]. A previous study demonstrated that the effective expression of *OLEOSIN* in *A. thaliana* requires the activation of two adjacent RY elements of the *LEC2* promoter [51]. LEC2 acts synergistically with ABI3 and LEC1 to enhance the activation of the *OLEOSIN* promoter in the developing embryo [52]. In summary, LEC2 regulates many genes that participate in different events and signaling pathways of early embryonic development and seed maturation (Figure 1).

The *LEC2* gene is involved in the regulation of both embryonic formation and maturation in *A. thaliana*. The heterologous expression of the *AtLEC2* gene in tobacco results in abnormal tobacco seedlings. Digital gene expression profile analysis has shown that the ectopic expression of the *AtLEC2* gene in tobacco could activate several genes and metabolic processes, including SSP, late embryogenesis abundant (LEA) protein, fatty acid biosynthesis, and sugar accumulation; in addition, *AtLEC2* can activate key regulatory genes such as *MADS-box protein 9*, *L1L*, *SERK1*, and *HAM*. The ectopic expression of *AtLEC2* affects the contents of stored substances and induces somatic embryogenesis in tobacco [53]. The latest research shows that the induced expression of *AtLEC2* could also trigger the formation of embryogenic calli in tobacco [54].

Castor bean is an essential oil crop that is capable of accumulating a large amount of TAG in its seeds. The *LEC2* gene identified in castor bean seeds is named *RcLEC2* and consists of six exons and five introns that are substantially homologous to the *LEC2* gene of *A. thaliana*. The heterologous expression of *RcLEC2* in *A. thaliana* induces the expression of related TFs that influence seed maturity, as well as the seed fatty acid biosynthesis gene *WRI1* (Figure 1), thereby resulting in an increase in TAG content [27]. The above results may facilitate the characterization of the regulatory mechanism of fatty acid and lipid synthesis during the growth and development of castor beans.

Similarly, three putative homologs of the *LEC2* gene in *A. thaliana* were identified in the monocot plant maize and were designated as *ZmAFL4*, *ZmAFL5*, and *ZmAFL6*. The *ZmAFL5* and *ZmAFL6* genes had the highest activity in ovules and kernels, and both genes exhibited constitutive gene reactivity. The *ZmAFL4* gene has preferential expression in corn tassels and pollen, and its expression profile is consistent with that of *LEC2*. The analysis of *ZmAFL4* gene expression in maize seeds indicated that its transcripts are abundant in the endosperm but are barely expressed in embryos, different from the active expression site of *LEC2* in *A. thaliana.* In addition, metabolomics analysis suggests that the reduction in *ZmAFL4* gene activity affects the carbon metabolism in corn kernels; the starch content of transgenic corn kernels at 20 DAP showed the most significant reduction. More importantly, *ZmAFL4* does not seem to be involved in the TFs that regulate maize seed storage proteins. These results indicate that the function of *LEC2* homologs of *A. thaliana* is not conserved, and no *LEC2* functional homolog has been found in monocots [13,55].

In soybeans, the *LEC2* homolog *GmLEC2a* regulates carbohydrate catabolism and triacylglycerol (TAG) biosynthesis; it also plays an important role in the development of plant seeds. Studies have shown that the ectopic expression of *GmLEC2a* in soybean hairy roots causes the upregulation of the *GmLEC1*, *GmFUS3*, *GmABI3*, *GmDof11*, and *GmWRI1* genes, which in turn enhance TAG biosynthesis. In addition, its ectopic expression also negatively regulated the expression of TAG lipase genes [56].

The finding that LEC2 TFs regulate lipids, sucrose, starch, oils, and proteins in various plant seeds of dicots indicates that LEC2 plays a major role in the maturation stage of seed development. Future research is warranted to understand how the *LEC2* gene participates in the regulation of various storage material synthesis pathways during seed maturation.

## 4. The *LEC2* Gene Plays a Crucial Role in Somatic Embryogenesis

Plant cells exhibit unique developmental plasticity that is related to totipotency. For example, the occurrence of somatic embryos is a good indicator of the pluripotency of plant cells. The formation of somatic embryos can be induced by treating cultured somatic cells with auxins and inducing the cells to differentiate in vitro [57]. Numerous studies have shown that the ectopic expression of TFs and their associated genes could induce spontaneous embryogenesis [58,59,60], among which the TF *LEC2* is instrumental in inducing the formation of somatic embryos.

In *A. thaliana*, the *LEC2* gene was cloned and expressed ectopically, and the results revealed that it was preferentially expressed during embryogenesis and that it has the ability to induce the formation of somatic embryos [12]. It has been suggested that auxin-induced plant somatic embryogenesis is a key factor in somatic embryo formation, i.e., LEC2 may affect the occurrence of somatic embryos by regulating auxin [61]. A previous study has suggested that *AtLEC2* could activate auxin in response to the expression of the *INDOLE-3-ACETIC ACID INDUCIBLE30* (*IAA30*) gene during embryogenesis [14]. Shortly afterward, when *LEC2* was ectopically expressed in *Arabidopsis* seedlings, somatic embryos were formed in the seedlings, and *AtLEC2* also activated the expression of the *YUCCA2* (*YUC2*) and *YUCCA4* (*YUC4*) genes for auxin biosynthesis [24]. The above findings imply that the ability of *LEC2* to induce the formation of somatic embryos may be derived from the activation of the *YUC2* and *YUC4* genes that mediate auxin biosynthesis, and that *LEC2* acts as a negative regulator of the auxin signal transduction-related *IAA30* gene [41,59]. Although the overexpression of *LEC2* in plants could induce the formation of somatic embryos, explants treated with auxin in vitro have produced damaged embryos. To better understand this phenomenon, *35S::LEC2-GR* transgenic explants were treated with different concentrations of auxin. The results demonstrated that *AtLEC2* augments endogenous auxin in the cultured explants, and the expression of three *YUCCA* genes (*YUC1*, *YUC4*, and *YUC10*) in the IPA-YUC auxin biosynthesis pathway related to somatic embryo induction also showed some correlation with *AtLEC2* [62]. Through RT-PCR analysis of the embryogenesis cultures of the explants described above, AtLEC2 was found to be a key regulator that could stimulate the transcription of the *YUC1*, *YUC4*, and *YUC10* genes. The overexpression of *AtLEC2* could also significantly upregulate the expression levels of these three genes when explants were cultured in an auxin-free medium. The increase in endogenous auxin is due to the activation of the *YUC* gene that regulates the presence and function of exogenous auxin. These findings provide an important perspective for the study of the LEC2-mediated formation of somatic embryos [63].

Regarding the fact that *LEC2* can induce somatic embryos, in addition to the possibility that it could regulate growth hormones, other hormones such as gibberellic acid and ethylene have been considered. Among these, ethylene, a gaseous plant hormone, participates in and controls the processes of plant growth and development [64,65]. Ethylene is regulated by *ERF022*, a gene that can induce effective embryogenesis in explants [66]. After mutating *ERF022* in *A. thaliana* seedlings, the content of ethylene was increased, and the ability of embryogenesis was reduced [67]. In a breakthrough report, researchers have documented that auxin–ethylene interactions are controlled by *ERF022* and *AtLEC2* and their targets during somatic embryo formation [66]. This provides information concerning the underlying mechanism by which *LEC2* regulates somatic embryo formation.

*AtLEC2* also influences somatic embryo formation in other plant species. Using the leaf disc method to transform *AtLEC2* into tobacco, embryogenic calli appeared on the stem apex meristems of tobacco [54], and then the structures of somatic embryos formed in the callus. This indicates that ectopic *AtLEC2* expression induces the formation of somatic embryos in tobacco [53]. Similarly, when *AtLEC2* was transferred to *Brassica napus*, somatic embryos with cotyledon-like and hypocotyl-like organ systems were formed on the cotyledon petioles, and their morphology and structure were similar to those of zygotic embryos [68].

Recent studies have shown that *TcLEC2* induces the formation of a large number of somatic embryos on the leaves of *Theobroma cacao* [69,70]. In cassava, an orthologous gene of *A. thaliana LEC2* has been identified and named *MeLEC2*. An analysis of the effect of its overexpression during somatic embryogenesis revealed that *MeLEC2* was unregulated in somatic embryos compared with differentiated mature plant tissues. Furthermore, qRT-PCR analysis has shown that *MeLEC2* plays a role in somatic embryogenesis in cassava. In addition, somatic embryogenesis, which is similar to zygotic embryogenesis, was observed in *MeLEC2* transgenic cassava leaves. This result demonstrated that *MeLEC2* has the ability to program vegetative cells to induce somatic embryogenesis [71].

In the legume *Medicago truncatula*, the *LEC2* gene has been identified and named *MtLEC2*. There are two near-isogenic types in *M. truncatula*; one is M9-10a with embryogenic ability, and the other was named M9 and had very low embryogenic ability [72,73]. The two genotypes of *M. truncatula* were introduced into leaflet explants in vitro and then detected by qRT-PCR during the formation of somatic embryos. The final results showed that the *MtLEC2* gene was highly expressed in the M9-10a explants, while the *MtLEC2* gene in M9 explants displayed a low level of expression. Expression profiling has shown that *MtLEC2* is involved in the occurrence of *M. truncatula* somatic embryos [31].

Taken together, these studies show that *LEC2* has a significant effect on plant somatic embryogenesis. However, further exploration of the regulatory effect of *LEC2* on somatic embryo formation at the molecular level is needed.

## 5. The Function of *LEC2* during Other Plant Developmental Stages

*LEC2* acts as the master regulatory factor in the processes of plant growth and development. It influences the occurrence of somatic embryos and also plays an important role in the growth phase of other plant structures. Studies have shown that *LEC2* is also closely related to the formation of lateral roots. LEC2 activated the transcription of the *NAC* gene family. NAC proteins play roles in plant developmental processes such as lateral root development [74]. *AtLEC2* also interacts with *AtFUS3* to activate the expression of the auxin biosynthesis gene *YUCCA4* (*YUC4*), which in turn promotes the generation of lateral roots in *A. thaliana* [29]. LEC2 and FUS3 have different binding sites in the *YUC4* promoter. They can both directly bind to different RY elements of the *YUC4* promoter. The FUS3–LEC2 interaction may enhance the ability of binding to RY elements to synergistically activate *YUC4* transcription. In the initial stages of lateral root formation, *AtLEC2* also activates *AtFUS3* expression [29]. The lateral root formation induced by LEC2 was partially attributable to the enhanced FUS3 expression.

In addition, *AtLEC2* could induce leaf reprogramming during development. The overexpression of the *LEC2* gene in *Arabidopsis* resulted in alterations of the morphological characteristics of leaves [26]. The leaves became smaller and curled, and developed into cotyledons. Furthermore, the leaves were less fleshy, and the number of trichomes was reduced. Based on the lack of research at the cellular level, this phenomenon could not be further analyzed. After the leaves were sectioned and stained with toluidine blue-O (TBO) dye solution, changes in the anatomical structure of the leaves were assessed. Leaf cells showed a tighter arrangement, and vacuoles were sharply decreased in number and were lightly stained by the dye solution [26].

When *LEC2* was overexpressed in the senescent leaves of *A. thaliana*, TAG content was augmented threefold compared with the wild type, and there were no negative effects on plant growth and development [75]. More importantly, *LEC2* upregulated the expression of multiple genes related to fatty acid and TAG synthesis in senescent leaves. Therefore, we deduced that *LEC2* regulates genes that are involved in the key metabolic steps of TAG and fatty acid synthesis, thereby greatly increasing TAG content in vegetative organs.

The wheat EST sequence was queried in the databases, and a homolog of the *A. thaliana* seed maturation regulator *LEC2* was detected and named *TaL2L* (*LEC2-LIKE*) [76]. Numerous *LEC2* orthologs have been identified in dicotyledonous plants, and *TaL2LA* was the first *LEC2* ortholog identified in monocotyledonous plants [76]. Researchers have reported that the *DELAY OF GERMINATION1* (*DOG1*) gene regulated seed dormancy in *A. thaliana* [77]. Several studies have suggested that the *DOG1* promoter contains the RY element. This implies that *DOG1* would be regulated by *LEC2* as in *Arabidopsis*, as the RY element is the target site of the TF, containing the B3 domain [11,14,77,78]. The germination index (GI) could be used to assess the influence of each TF in seed dormancy. In wheat, except for the dormant cultivars, the expression level of *TaL2LA* is significantly correlated with the GI of seed dormancy levels. The expression of *TaDOG1* in wheat is also significantly correlated with seed dormancy, suggesting that *TaL2LA* influences wheat seed maturity and dormancy by regulating the expression of the *TaDOG1* gene [76].

The cellulose synthase 8A protein (PtdCesA8A) of poplar trees is highly expressed in the xylem cells of poplar trees [79] and also shows high activity in the xylem of transgenic tobacco plants. The *A. thaliana DGAT1* and *LEC2* genes are linked to the xylem-specific promoter *PtdCesA8A*, and these were transformed into transgenic tobacco plants. The results demonstrated that a large supply of fatty acids and TAGs accumulate in the stems of tobacco, and no abnormalities in the growth and development of the plant were observed [80].

The above results indicate that the *LEC2* gene plays a regulatory role in other developmental processes of different plant species, indicating the versatility of the *LEC2* gene. However, LEC2 is expressed from the early globular stage until the early torpedo stage. Although LEC2 has been shown to control *OLEOSIN* genes, its pattern does not fit with the late embryogenesis and heavy fat reserve accumulation that are the foci of ABI3 regulation. The current research is focused on the biological significance and regulatory molecular mechanisms underlying these biological processes.

## 6. Conclusions and Future Perspectives

The molecular mechanisms that affect plant growth and development regulatory networks have been explored in depth. Among these, the functional expression of the *LEC2* gene in plants has resulted in significant progress in plant biology and the application of biotechnology to transgenic plants.

Studies in the model plant *A. thaliana* indicate that *LEC2* is the main regulatory factor involved in the growth and development of plant seeds. The expression of *LEC2* can induce the occurrence of somatic embryos throughout the development process. Various lines of evidence indicate that the expression of *LEC2* is involved in important biological processes such as embryogenesis, synthesis of storage proteins, fatty acids, and TAGs, as well as in the formation of lateral roots during seed growth and development. *LEC2* is not only a direct transcription activator but also acts as a leading TF gene in various plant species. *LEC2* participates in a variety of signaling pathways and regulates the expression of multiple key genes. The above research results demonstrate that the functions of the TF LEC2 are diverse and are important in many aspects of plant development.

In addition, the orthologs of the *Arabidopsis LEC2* gene and the characterization of their functions have been identified in crops such as maize and wheat. Although the *LEC2* orthologs identified in cassava and soybeans show functional conservation, these have certain differences compared to the orthologs in maize, allowing us to better understand the functions of *LEC2* homologs in different species. At the same time, the discovery of orthologs of the *LEC2* gene in wheat could facilitate a better understanding of the process of regulating seed dormancy. The above studies improve our understanding of the conservation and functional differences of *LEC2* genes in different plant species and provide us with a broader perspective of the functions of the *LEC2* genes.

However, there are still many problems to be further explored and solved. For example, in addition to embryological processes, does *LEC2* regulate other unknown biological processes in plant development? Continuous in-depth research on *LEC2* may result in the identification of new signaling pathways and thus improve our understanding of the biological function of *LEC2*.

## Figures and Tables

**Table 1:** Biological function of LEC2 transcription factor in *A. thaliana*.

Biological Function	Reference
Induces somatic embryos and embryonic development in vegetative cells	[4]
Initiates somatic embryo development	[12]
Regulates the expression of storage protein genes	[11]
Induces somatic embryogenesis	[22]
Triggers the stockpile of oil and seed-specific mRNAs	[23]
Induces maturation traits and auxin activity	[24]
Affects the contents of oil and protein, starch, and sucrose	[25]
Changes the shape and anatomy of leaves	[26]
Triggers the expression of genes encoding seed maturation and oil body protein regulators in trophic organization	[27]
Promotes embryogenic callus formation in roots	[28]
Controls the formation of lateral roots	[29]
Involved in early embryogenesis Participates in the development of somatic embryos	[30] [31]

## Data Availability

Not applicable.

## Abbreviations

LEC2	LEAFY COTYLEDON 2
ABI3	ABA INSENSITIVE3
FUS3	FUSCA3
LEC1	LEAFY COTYLEDON1
TAG	Triacylglycerol
SSP	Seed storage protein
AGL15	AGAMOUS-LIKE15
HAM	Hairy meristem
IAA30	Indole acetic acid inducible 30
SUS	Sucrose synthase
LEA	Late embryogenesis abundant
DOG1	DELAY OF GERMINATION1
